# Water and Aqueous Mixtures as Convenient Alternative Media for Organoselenium Chemistry

**DOI:** 10.3390/molecules21111482

**Published:** 2016-11-06

**Authors:** Claudio Santi, Raquel G. Jacob, Bonifacio Monti, Luana Bagnoli, Luca Sancineto, Eder J. Lenardão

**Affiliations:** 1 Department of Pharmaceutical Sciences, Group of Catalysis and Organic Green Chemistry, University of Perugia, Via del Liceo 1, Perugia 06100, Italy; bonifaciomonti@gmail.com (B.M.); luana.bagnoli@unipg.it (L.B.); sancineto.luca@gmail.com (L.S.); 2 Laboratório de Síntese Orgânica Limpa-LASOL-CCQFA-Universidade Federal de Pelotas-UFPel, P.O. Box 354, Pelotas 96010-900, RS, Brazil; raquel.jacob@ufpel.edu.br (R.G.J.); elenardao@uol.com.br (E.J.L.)

**Keywords:** on-water reactions, water, selenium, epoxide, zinc, michael addition, coupling, catalysis, oxidation

## Abstract

Even if water is the natural environment for bioorganic reactions, its use in organic chemistry is often severely limited by the high insolubility of the organic derivatives. In this review, we introduce some examples of the use of water to perform organoselenium chemistry. We mainly discuss the advantages of this medium when the recyclability is demonstrated and when the water can control the selectivity of a reaction or enhance the reaction rate.

## 1. Introduction

During the last two decades, the use of water as an alternative solvent for organic reactions has been debated. Water is the natural medium for all biochemical reactions and is often claimed as an ideal green solvent [1,2] because it is non-toxic and non-flammable, has a high heat capacity in adsorbing the energy produced during a chemical reaction, and has low cost and large availability. Breslow [3], Sharpless [4], and several other authors [5] reported reactions that can be efficiently conducted “in-water” or “on-water” conditions. This latter term describes those reactions that when performed in vigorously stirred aqueous suspension showed a remarkable rate acceleration, only partially due to the well-known hydrophobic effect [6], which, on the contrary, has been demonstrated to be responsible for the selectivity observed in some biomimetic protocol performed using water as a reaction medium [7,8,9,10,11]. Despite the low solubility or complete insolubility of organic molecules, water presents several unique physical properties that can affect the reactivity and selectivity, such as hydrogen bonding, acidity, polarity, and entropy.

On the other hand, in most cases, large amounts of organic solvents need to be used to extract the organic compounds from the water. It was reported that for several organocatalytic reactions the amount of organic solvents used in the workup exceeds the volume of water used as medium by a factor of 30-fold. The result is that in those cases the water recovered as waste is contaminated by toxic elements, representing a limitation for its use as a green alternative solvent [12].

A more realistic consideration suggests that a complex set of parameters needs to be critically considered to affirm or debunk the environmental and economic convenience of the use of water in organic reactions. Probably it is not misleading to make a generalization but it is much more correct to analyze case by case. At the same time, in several examples the selectivity, as well as the reactivity or the possibility of recovering the catalyst, the reaction media, or the final product with simple and economic procedures represent interesting aspects to consider during the design of a chemical reaction that can suggest the use of water as alternative solvent or medium.

In this review, we will not describe the use of water simply as a non-conventional green solvent for organochalcogen reactions. This aspect was recently stressed by Perin in the context of a wider review that deals with all the unconventional reaction media for organochalcogen chemistry [13]. In the present manuscript we directed our attention to a series of advantages and disadvantage that can be correlated to the use of water or aqueous conditions for reactions involving organoselenium reagents with particular emphasis on some examples in which the aqueous medium can be easily recovered and reused, contributing to the sustainability of the reaction by reducing the production of waste and enabling an easy turnover of the catalyst. This review is not an exhaustive collection of all the data reported in literature but a selection of examples that clarify our ideas on the topic.

## 2. Nucleophilic Organometallic Selenium Reagents

Even if water is commonly used as a reactant for the electrophilic hydroxyselenenylation of double and triple bonds [14], to the best of our knowledge the first examples reporting its use as medium referred to the formation and the reaction of nucleophilic species of selenium mediated by metals. Organometallic reactions in aqueous conditions have attracted the interest of several research groups in the recent past [15], even if the use of this medium can be considered uncommon since almost all the well-known organometallic reactions (Grignard, Barbier, Reformatsky, Gilman, etc.) are moisture-sensitive and are normally carried out under inert conditions. In 1973 Klayman et al. reported the use of protic solvents for the reaction of elemental selenium with sodium borohydride as a convenient method to introduce this element in organic substrates [16]. On the basis of the Se/NaBH_4_ ratio, the authors claimed the formation of NaHSe (for a Se/NaBH_4_ ratio = 1:2) or Na_2_Se_2_ (for a Se/NaBH_4_ ratio = 1:1) in water. When the protic solvent was methanol, the borohydride was partially decomposed and the protocol required a larger amount of reductant to promote the complete dissolution of the chalcogen and the formation of sodium hydrogen selenide or sodium diselenide, respectively.

**Figure 1:**
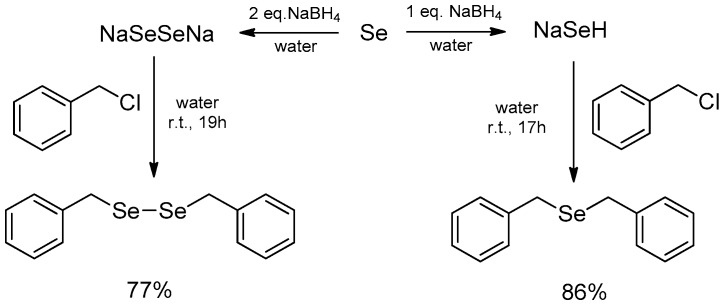
Water as a protic solvent for the synthesis of selenides and diselenides.

The reaction of benzyl chloride with aqueous solution of both the abovementioned reagents produced, after several hours at room temperature, the corresponding dibenzyl selenide (86% yield) and diselenide (77% yield), as depicted in Figure 1. This protocol represents an interesting alternative to a very old procedure that, using an alkaline aqueous solution of Rongalite^®^ (hydroxymethansulfinate), provided the synthesis of NaSeNa [17].

**Figure 2:**
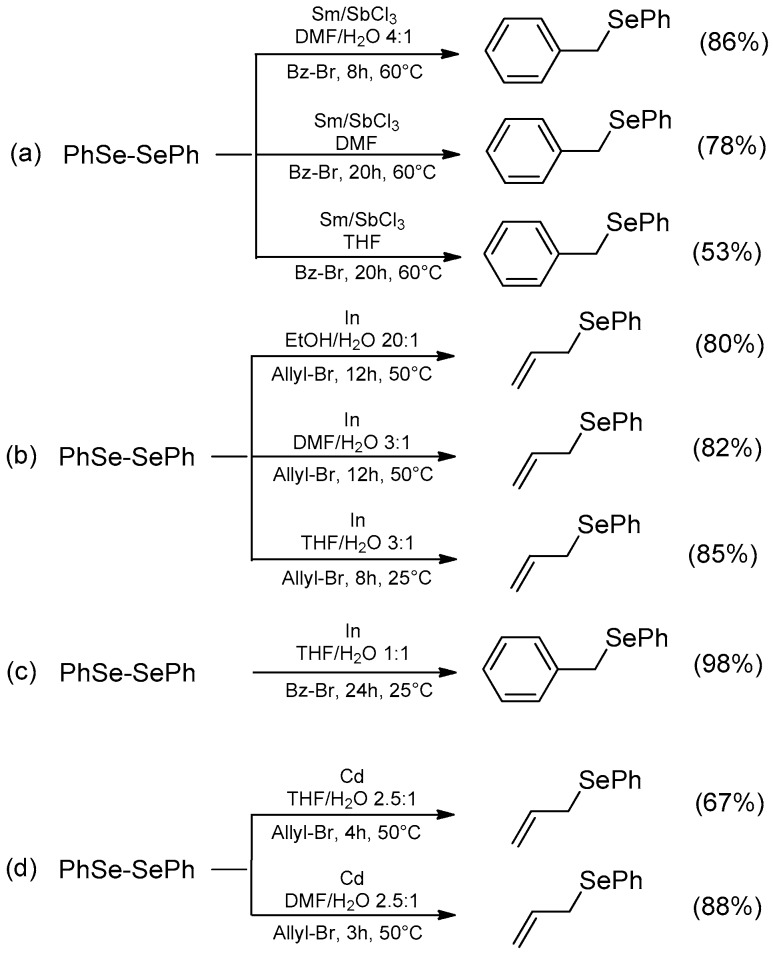
Nucleophilic selenenylation of benzyl and allyl bromides: (**a**) promoted by Sm/SbCl_3_; (**b**,**c**) promoted by Indium; (**d**) promoted by Cadmium.

The selenium-selenium bond can be reductively cleaved in the presence of several metals; this represents an interesting strategy to introduce an organoselenium moiety in organic substrates having suitable electrophilic properties. Some of these reactions were carried out in the presence of water as a co-solvent, showing an interesting improvement in reactivity in terms of yields and reduced reaction time when compared with other organic solvents (Figure 2).

The couple Sm/SbCl_3_ (Figure 2a) produces in situ metallic antimony, which, based on the mechanism proposed by the authors, produces a benzylantimony intermediate by the reaction with benzyl bromide and then reacts with the diselenide, even if, in our opinion, a direct oxidative insertion to the Se-Se bond cannot be excluded [18]. The reaction was carried out at 60 °C in anhydrous THF, anhydrous DMF, and a mixture of DMF/water (4:1), producing, in this latter case, a considerable improvement in terms of reaction rate and yield.

Similarly, indium has been used to synthesize allyl (Figure 2b) and propargyl selenides in aqueous media. The authors reported the combination of water with different organic solvents such as EtOH, DMF, and THF, proving that in a 3:1 mixture of THF and H_2_O the reaction is considerably faster, giving up to 85% yield in 8 h compared to the 12 h required for other aqueous media [19] and anhydrous THF [20]. Later, Galindo et al. confirmed this procedure (Figure 2c), reporting that in THF the reaction produced the desired product in lower yields (not indicated in the paper) [21]. The same authors speculated about the mechanism, proposing that a general interpretation can be summarized by the triad reaction mechanism that ranges between a radical process, a covalent C–M (or Se–M) activation and an anionic process. Case by case the prevalence of one of these mechanisms is determined by the nature of the substrate, by the metal, and by the reaction conditions [22].

Metallic cadmium, generated in situ by the reduction of CdCl_2_ with samarium, has been reported for the selenenylation of allyl bromides (Figure 2d) and α-bromocarboxylates. The reaction proceeds in aqueous conditions, giving better results in 2.5:1 DMF/water with respect to 2.5:1 THF/water mixtures [23]. Similar results were obtained using the couple Sm/BiCl_3_ in 4:1 DMF/water [24] and elemental tin in a 10:1 THF/water mixture [25].

**Figure 3:**
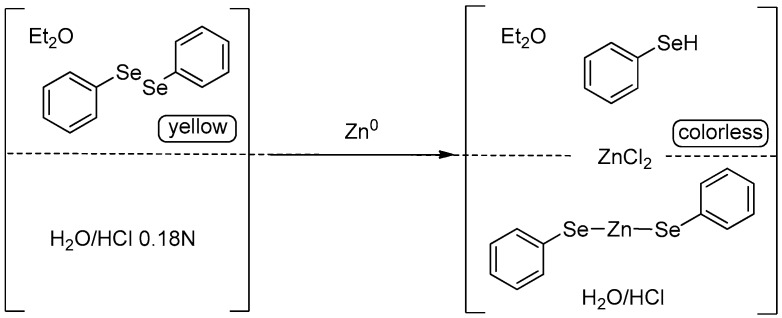
Reduction of diselenides in a biphasic aqueous system.

More recently, some of us introduced a simple procedure to reduce diselenides with elemental zinc in a biphasic system composed of diethyl ether and a water solution of HCl (0.18 N). With diphenyl diselenide, the discoloration of the organic phase indicates the complete cleavage of the Se-Se bond and the formation of a nucleophilic reagent(s) that is most probably composed of a mixture of PhSeH and PhSeZnSePh [26] (Figure 3).

These reactions are carried out in closed vials and the prevalent formation of the complex prevents the bad smell typical of selenols. In addition, the protocol was demonstrated to be applicable to a broad range of diselenides. Several electrophiles can be added directly to the biphasic system, producing the corresponding selenylated derivatives that can be easily extracted by separation of ethereal and aqueous phases.

**Figure 4:**
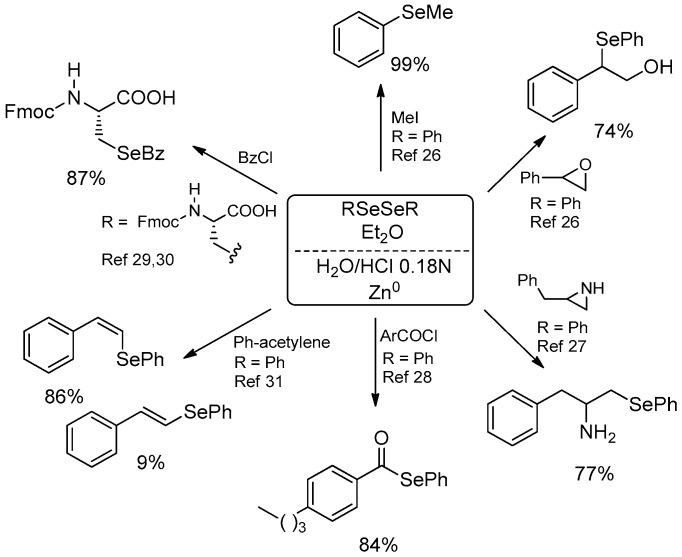
Synthetic applications of the zinc selenate prepared in the biphasic system.

Some representative examples are reported in Figure 4. The reduced mixture reacted with aliphatic halides, producing variously structured selenides; the fact that primary halides reacted faster than tertiary ones clearly indicated that the mechanism does not involve a radical pathway [26]. Similarly, a broad range of epoxides was reacted, leading to the corresponding β-hydroxyselenides. The acidic conditions in the case of phenyl substituted epoxides forced the regioselective attack of the selenium atom to the more hindered carbon atom through the intermediate formation of a carbocation or an incipient positive charge formed in the benzylic position [26].

An interesting application of this procedure was reported by Braga and coworkers, who described the synthesis of a series of chiral β-seleno amines by the ring-opening reaction of unprotected aziridines. The acidic biphasic system is fundamental to activate the substrate by protonation of the nitrogen atom, obtaining good yields with aliphatic and aromatic diselenides. An example of tellurium derivative was also reported. The biphasic protocol allowed the use of simply available unprotected aziridines, avoiding the need for expensive protection and deprotection synthetic steps [27].

**Figure 5:**
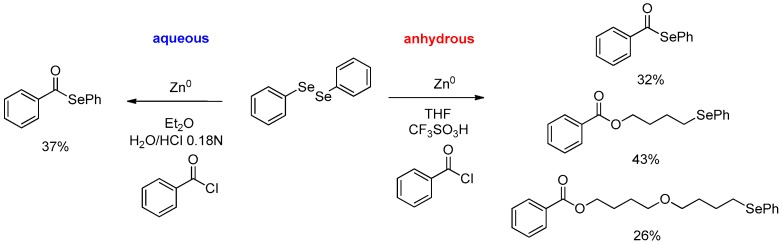
Chemoselectivity in aqueous conditions.

Surprisingly, acyl chlorides could also be treated in the acidic biphasic system, producing selenoesters in moderate to good yields. The acyl substitution, probably due to the presence of a zinc atom that coordinates the carboxylic moiety, brings the selenium atom nearer to the electrophilic carbon, facilitating the competition of acyl substitution with respect to the most predictable hydrolysis [28]. Is worth mentioning that the use of the biphasic water/diethyl ether system prevents a side reaction that was recently observed when the oxidative zinc insertion and the subsequent reaction with benzoyl chloride were performed in refluxing THF (using trifluoroacetic acid or triflic acid as catalyst). In these circumstances the ring-opening reaction of the solvent competes with the acyl substitution, producing selenoderivatives in which the selenium moiety and the carboxylic one are spaced by four carbon units [28] (Figure 5).

Flemer recently reported the synthesis of cysteine (Cys) and selenocysteine (Sec) derivatives as building blocks in the preparation of chalcogen-containing peptides [29]. Several derivatives were prepared starting from bis-protected dichalcogenides and the author stated that the biphasic protocol results were superior to almost all the other reduction methods reported in the literature for the reductive cleavage of the chalcogen-chalcogen bond [30].

**Figure 6:**
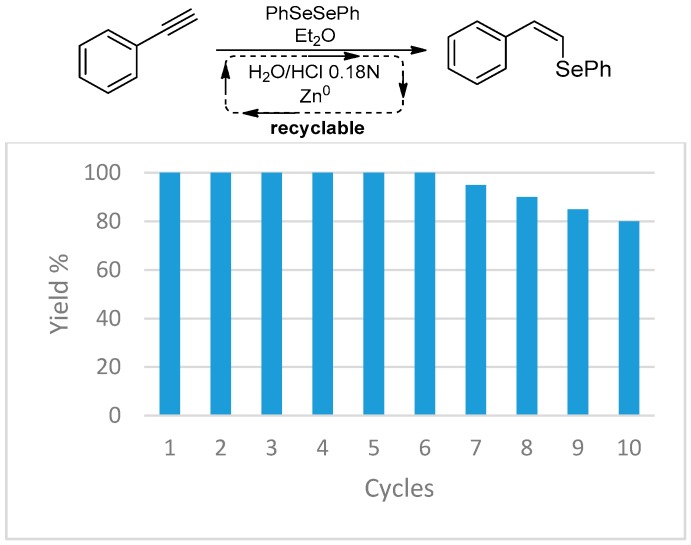
Yield of the hydrochalcogenation of phenylacethylene for 10 cycles, reusing water and unreacted zinc.

Starting from alkynes, vinyl selenides and vinyl sulfides can be easily prepared by hydrochalcogenation with selenols and thiols generated in situ by the reduction of the corresponding diselenides and disulfides in the easily recyclable biphasic system. It was demonstrated that the metal is not a mere spectator but forces the selectivity of the reaction in favor of the *Z* isomer [31]. In this reaction, the possibility of reusing the aqueous phase together with the unreacted zinc up to 10 times while maintaining appreciable yields represents an interesting improvement on the greenness of the process, preventing waste production (Figure 6).

An interesting class of bench stable zinc selenates was synthesized through oxidative insertion of the elemental zinc into the selenium–halogen bond of commercially available PhSeCl and PhSeBr. The resulting zinc complexes showed an unusual stability in non-inert conditions and, more interestingly, a considerable rate acceleration when the nucleophilic selenenylation reactions are performed under “on-water” conditions [32]. As an example, PhSeZnCl was used for the synthesis of alkyl phenyl selenides from the organic alkyl halides and when the reaction was carried out in water suspension the reaction time was 10–12-fold shorter than in THF solution. Interestingly, the stability of this reagent was also higher in water than in THF; in this latter case light-activated decomposition to produce the corresponding diselenide was completed within a few hours.

Primary halides gave better yields compared to the secondary one and, starting from the tertiary halides, no reaction was observed. This indicates that the reaction is an S_N_2 nucleophilic substitution and radical species are not involved in the mechanism.

Bieber et al. previously reported the same reaction using diphenyl diselenide and zinc in an aqueous medium. Nevertheless, in this case the reaction required acidic or basic conditions and the presence of an organic co-solvent (MeCN in a 1:2 ratio with water) in order to promote the reduction and overcome the non-solubility of the diselenide. The authors reported evidence of the involvement of alkyl radical species and proposed an S_H_2 mechanism to explain all the observed results [33].

**Figure 7:**
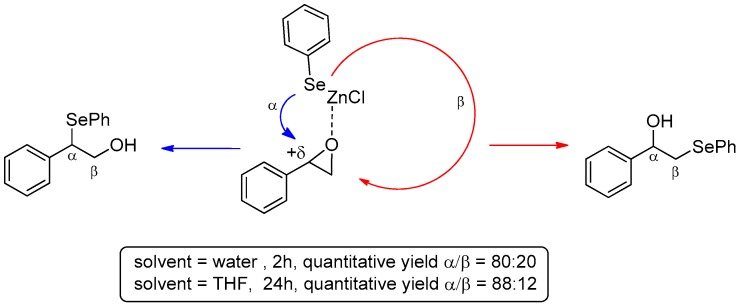
Regioselectivity in the ring opening of epoxide with of PhSeZnCl.

In parallel with the investigations carried out using the aqueous biphasic system, PhSeZnCl has also been used for the ring opening of several epoxides. In this case, it is worth mentioning that even if the reaction conditions are neutral the presence of the -ZnCl group confers to the molecule a Lewis acid character, which is enough to drive the regioselectivity of the ring opening of styrene oxide, leading the formation of the β-hydroxyselenide arising from the ring opening to the more hindered benzylic position (carbon α). The use of “on-water” conditions strongly accelerated the reaction, giving selenylated alcohols in good yield after 2 h at room temperature, compared with the 24 h required in THF. On the contrary, a moderate loss of regioselectivity was observed in water, probably because the non-solubility reduces the Lewis acid character of the reagent, disfavoring the interaction of the zinc with the oxygen atom of the heterocycle (Figure 7).

The opposite regio-selection can be obtained in water using benzenselenol as nucleophile under the supramolecular catalysis of β-cyclodextrins. Reaction times are longer (24 h) in comparison with the similar reactions reported above (2 h) but extremely mild conditions are required to avoid acidic or basic activations as well as catalysis with heavy metals. In addition, the use of water as a medium allowed the easy and almost complete reuse of the cyclodextrins [34].

**Figure 8:**
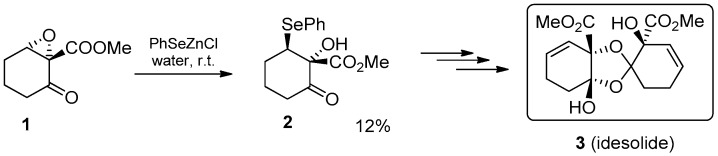
PhSeZnCl in the total synthesis of idesolide.

PhSeZnCl in “on-water” conditions was applied in a step of the total synthesis of *idesolide* (**3**) [35], a metabolite of *Idesia polycarpa*, studied as an inhibitor of apoptosis [36]. The ring opening of the epoxy keto ester **1** produces the β-selenoalcohol **2**, allowing the introduction of a carbon–carbon double bond by oxidative elimination of the phenylseleno moiety and the formation of the corresponding allylic alcohol. Even if the reaction resulted in low yields (12%), PhSeZnCl was comparable or superior to all the other tested methods, including the classical Sharpless methodology [37] (PhSeSePh, NaBH_4_ in MeOH), which produced a complex mixture of unidentified compounds (Figure 8).

Only a few examples of the ring opening of aziridines were reported using zinc selenates; apart from the examples reported above, in the biphasic system no experiment was performed in “on-water conditions.” As a general consideration, Braga et al. demonstrated that PhSeZnBr is generally more efficient than the corresponding chloride and that the best reaction medium is (BMIM)BF_4_. The authors also demonstrated that the ionic liquid can be easily recovered and reused at least four times [38].

The first example of Michael-type addition of benzenselenol to conjugate alkene in water was reported by Rao et al. in 2009 in the presence of cyclodextrins that are able to complex the selenol and the electrophile facilitating the reaction. The scope reported evidenced a broad applicability, giving good yields (ranging from 80% to 88%) starting from alkenes conjugated with ketones, aldehydes, esters, amides, and nitriles with long reaction times at room temperature (20–45 h depending on the substrate) [39]. Similarly, PhSeZnCl reacts with α,β-unsaturated ketones, producing in THF the corresponding selenides in longer time and comparable yields. Worth mentioning is that, in this case, when the reaction was carried out in “on-water” conditions no rate acceleration was observed and the reaction was considerably longer, as expected considering the low solubility of the reactants without any contribution of the hydrophobic effect. In addition, due to the instability of the zinc selenate in organic solution all the reactions need to be performed in a dark vial to avoid photo-activated decomposition. As an example, the conversion of cyclohexen-2-one (**4**) into the selenide **5** is reported in Table 1 [40].

**Figure 9:**
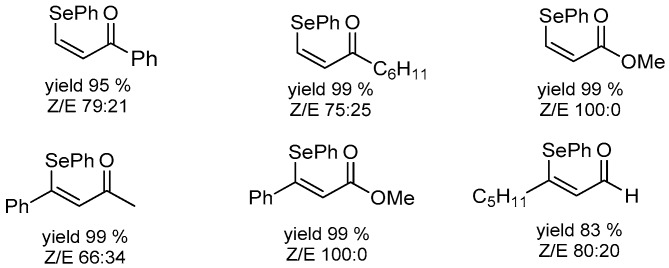
Vinyl selenide prepared by conjugated addition of PhSeZnCl to propargylic derivatives in “on-water” conditions (r.t., 2 h).

Completely different is the reactivity of PhSeZnCl toward Michael acceptors containing a carbon-carbon triple bond. The reaction is strongly accelerated by water (2 h vs. 24 h in THF) and the scope is broader, including aldehydes and esters as well as ketones, which produces the corresponding vinyl selenides in good yield and high stereoselectivity in favor of the *Z* isomer. Some examples are collected in Figure 9. The different behavior suggests a different mechanism between conjugated alkenes and alkynes in which the water plays a role in the activation of the substrate rather than the reactant.

**Figure 10:**
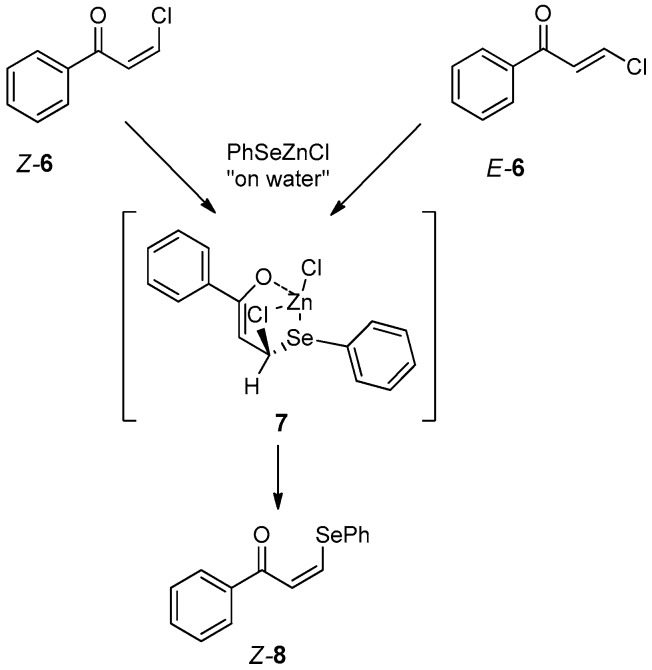
Vinyl substitution with a stereo-convergent mechanism.

Vinyl selenides can be obtained from PhSeZnCl using water as a reaction medium also toward vinyl substitution of the corresponding vinyl halides. The reaction in water is considerably faster than in THF and in all cases a stereo-retention on the geometry of the double bond was observed. Only in the case of the electron-deficient vinyl chloride **6** did the substitution from *Z* and *E* isomer occur with a stereo-convergent mechanism, producing *Z*-**8** as a unique or major isomer. This aspect has been investigated using DFT calculation, which shows that the substitution passes through the intermediate **7** (deriving from a sort of Michael-type addition), starting from both the *E*-**6** and *Z*-**6** isomers (Figure 10) [41].

The acyl substitution using zinc selenates to synthesize selenoesters was recently reported by different authors using non-conventional reaction conditions. Braga et al. optimized a solvent free protocol in which the selenenylating species (supposed to be PhSeZnSePh) is generated in situ by the reduction of the selenium-selenium bond of diphenyl diselenide with elemental zinc under microwave activation (100 W) and reacted in a one-pot manner with the appropriate acyl chloride [42]. Alternatively, the bench stable PhSeZn-halides were used either in solvent-free condition, under mechanochemical activation [43] or in “on-water” conditions at room temperature for 3 h [44]. In Table 2 these different protocols are compared based on the conversion of benzoyl chloride **9** into the corresponding selenoester **10**. It is evident that the “on-water” condition is able to efficiently activate the reaction without the need to use microwave, mortar, or conventional heating. In these conditions, PhSeZnBr was more effective than the analogous chlorine, probably as a consequence of the higher characteristic of Lewis acid.

**Figure 11:**
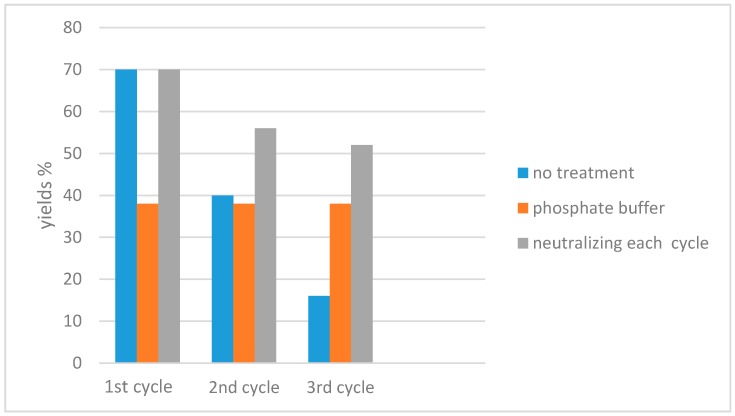
Recyclability of the aqueous medium for the conversion of **9** into **10** using the conditions depicted in Table 1 entry 6.

In addition, when the reaction was performed in water it was possible to recover and reuse the medium at last three times, increasing the greenness of the proposed methodology. The recyclability of the medium needed a treatment of the water in order to control the acidity generated during each reaction. In particular, reusing the medium caused the yields to drop dramatically after the first cycle. The use of a phosphate buffer produced moderate, if constant, yields in the three reactions, while the addition of sodium carbonate to neutralize HCl allowed good yields to be obtained in each cycle (Figure 11).

The presence of zinc was hypothesized [43] and later proven by DFT calculation [44] to be responsible for the unexpected ability of the selenium to compete with water in the attachment to the carboxylic carbon, favoring the nucleophilic acyl substitution over the hydrolysis. The coordination of the oxygen with the zinc (of the PhSeZnX) that acts as a Lewis acid activates the carbon toward the nucleophilic attack, bringing the selenium nearer to the electrophilic carbon and allowing the desired reaction to be faster with respect to the hydrolysis.

## 3. Water as an Alternative Medium for Organoselenium-Catalyzed Reactions

Even if the use of inorganic selenoxide (SeO_2_) for the catalytic allylic oxidation of olefins was reported by Sharpless in 1977 [45] and it was later demonstrated that the same catalyst, in the presence of hydrogen peroxide as an oxidant in a mixture of water and dioxane, can convert olefins into the corresponding diols [46], it was only in 2009 that the use of organoselenium catalysts was, for the first time, claimed as a convenient, green alternative to conventional stoichiometric protocols or metal-catalyzed reactions [47]. These opened the way for a series of applications designed in order to address the principle of Green Chemistry, and a number of interesting results obtained during the last five years were recently collected in a review article [1].

**Figure 12:**
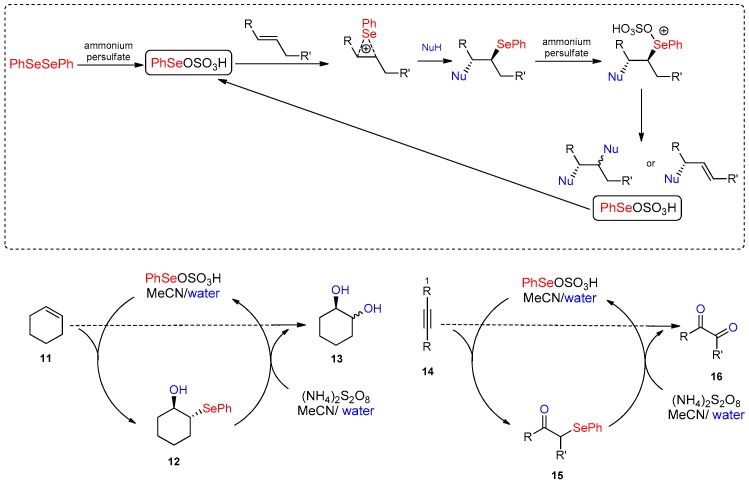
One-pot catalytic dihydroxylations.

The use of water in the electrophilic functionalizion of carbon–carbon multiple bonds promoted by selenium reagents is a well-known way to introduce a hydroxyl or amido group as the nucleophilic counterpart of the selenium in the attack to an olefin [48]. When the electrophile is generated in situ by the oxidation of a diselenide with ammonium persulphate, an excess of oxidant can activate the selenium moiety of the phenylselenide intermediate (**12**, **15**) toward a deselenation process that occurs by elimination or substitution, depending on the reaction conditions and the nature of the substrates. From the deselenation the electrophilic species is regenerated, allowing a catalytic role of selenium. When the substrate cannot stabilize the elimination product by conjugation and in the presence of a nucleophile (such as the water used as reaction medium), the selenonium salt (formed by the oxidation of the selenide intermediate) is replaced by a hydroxyl group, producing vicinal diol **13** from alkenes **11** and glyoxylic derivatives **16** from alkynes **14** [49,50] (Figure 12).

By contrast, when the oxidant is hydrogen peroxide the selenium-catalyzed reaction did not proceed via the electrophilic addition of selenium but toward an oxygen transfer mediated by the peroxyseleninic acid originating from the oxidation of the diselenide. Starting from electron-poor aryl diselenides, it was possible to convert olefins into the corresponding epoxides using perfluorinated alcohols as the solvent [51,52]. Nevertheless, when using a 30% aqueous solution of hydrogen peroxide it is impossible to avoid the formation of a moderate amount of the corresponding diols generated by an undesired ring opening reaction of the epoxide.

**Figure 13:**
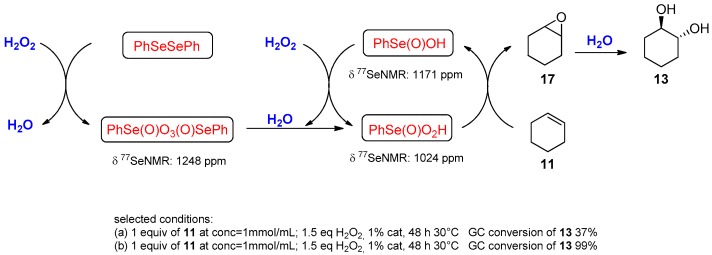
Oxidation of olefins mediated by PhSeO_3_H.

Based on this consideration, it can be envisioned that the presence of water as a reaction medium can be used as an efficient strategy for the oxidation of alkenes into the corresponding vicinal diols. The intermediate formation of an epoxide confers to the dihydroxylation a *trans* stereoselectivity that was reduced in cases in which the ring opening can occur via carbocation. A detailed study of the conversion of cyclohexene into the corresponding anti-1,2-diol was reported in two different papers by Yu et al. Following the reaction by GC, they reported that, in the presence of a slight excess of hydrogen peroxide in acetonitrile at 30 °C, 1% of diphenyl diselenide almost completely converts the cyclohexene **11** into the corresponding anti-diol **13**. The authors also reported that the success of the reaction strongly depends on the concentration of the starting material and the reagents. Higher concentrations correspond to higher yields, indicating that most probably a side reaction with the organic solvent can consume the peroxide, negatively affecting the final results [53]. Later the same authors, comparing the reactivity of a number of selenium catalysts using the same methodology, found that 1,2-bis(3,5-bis(trifluoromethyl)phenyl)diselane was the most efficient in terms of yield and reaction rate. In addition, they demonstrate by NMR spectroscopy that in the reaction mechanism the oxygen transfer species, when the precatalyst was diphenyl diselenide, is peroxyseleninic acid [54] (Figure 13).

**Figure 14:**
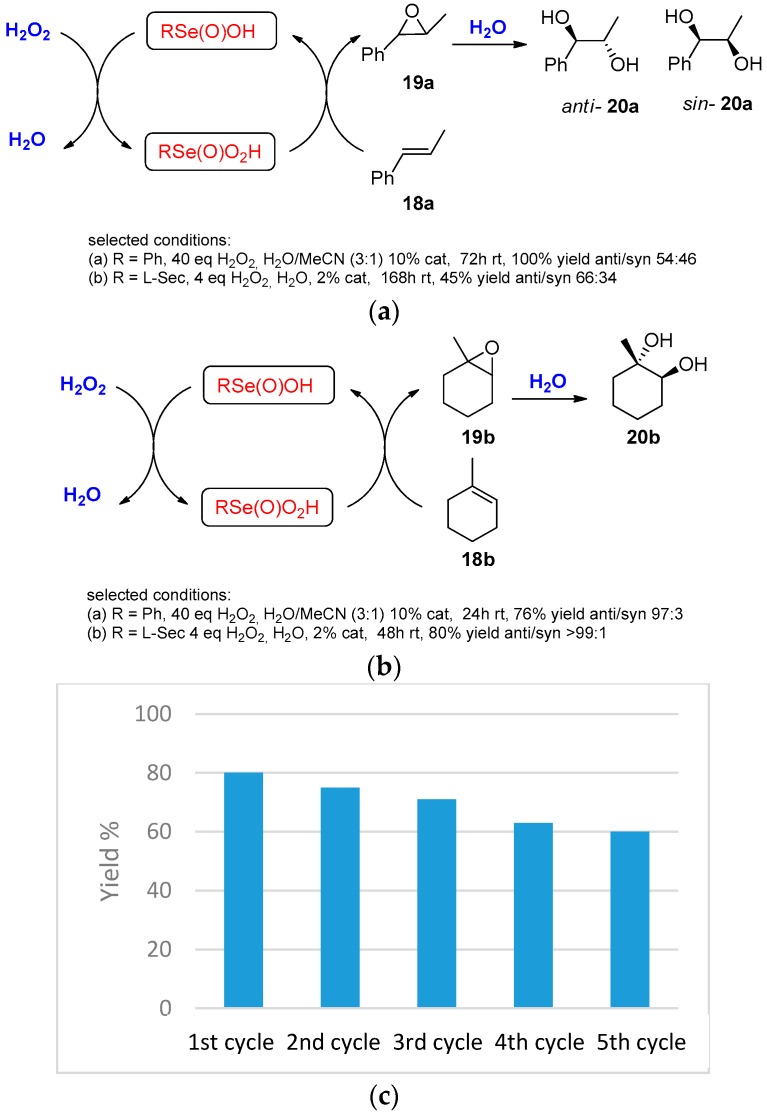
(**a**) and (**b**) Selenium catalyzed oxidation of **18a,b** into **20a,b**; (**c**) Recyclability of the catalyst and the water in the oxidation of **18b** into **20b**.

Previously, it was reported that similar reactions can be performed in a water/MeCN (3:1) mixture using 10% of diphenyl diselenide [55] or water using 2% of selenocysteine at room temperature (Figure 14) [56]. The latter was slightly better in terms of stereoselectivity but considerably slower, probably as a consequence of the reduced loading of the catalyst. The selectivity depends on the nature of the substrate and is moderate in the case of aryl substituted olefins and good in the case of the alkyl-substituted one, producing, in the case of methyl cyclohexene, only the *anti*-isomer in 80% yield and 80% of facial selectivity. Interestingly, when LSec)_2_ is the catalyst, after the extraction of the organic components, the aqueous phase and the dissolved catalyst can be recovered and reused after the addition of one equivalent of oxidant for five subsequent cycles with only a slight decrease of yields. When the reaction was performed in methanol, the epoxide intermediate evolved toward regio-specific formation of the corresponding hydroxymethoxylation product in excellent yield (93%) but a considerably lower stereoselectivity (37%). This was attributed to the presence of a side non-catalyzed reaction that in water can be controlled and thus avoided.

The current catalytic species was investigated by NMR, evidencing a peak on ^77^Se-NMR that clearly indicates an overoxidized species of selenium (1204 ppm) that was attributed to the peroxyseleninic acid and that, in light of the evidence reported later by Yu et al., could also be assigned to the corresponding peroxyanhydride [54].

Shaldon et al. reported that Baeyer-Villiger reactions can be efficiently carried out with aqueous hydrogen peroxide as the oxidant at room temperature using as a catalyst the 3,5-bis(trifluoromethyl)benzeneseleninic acid. The reaction required 1,1,1,3,3,3-hexafluoro-2-propanol, 2,2,2-trifluoroethanol, or dichloromethane as solvents and resulted in the formation of lactones (esters) or carboxylic acid, starting from ketones or aldehydes, respectively [57]. In addition, the same authors found that 3,5-bis(perfluorooctyl)benzeneseleninic acid can efficiently catalyze the oxidation of aldehydes as well as ketones using an aqueous solution of hydrogen peroxide in monophasic, fluorous biphasic, or triphasic reaction media [58]. In 2015 some of the present authors demonstrated that benzeneseleninic acid under “on-water” conditions oxidizes a number of aldehydes into the corresponding carboxylic acids when the medium is water or, alternatively, into the corresponding esters when the medium is an alcohol [59]. Almost all the carboxylic acids are not soluble in water, thus they can be easily isolated simply by filtration, recovering the aqueous phase that contains the catalyst that was reused for subsequent cycles. Using this procedure, a gram-scale synthesis and purification of benzoic acid from benzaldehyde was performed with an overall yield of 87%, evidencing after crystallization the total absence of selenium in the final compound. Several aspects of this reaction can be highlighted as addressing the principles of Green Chemistry: (1) the aqueous medium allowed us to avoid organic solvents both as reaction medium and for purification procedures, (2) the use of a stoichiometric amount of hydrogen peroxide increases the atom economy and the safety of the process mainly for large-scale production, and (3) the possibility of recycling and reusing the catalyst and the medium at least five times without any further treatment allows a reduction of the waste produced from the synthesis and the workup.

## 4. Other “On-Water” Conditions for the Synthesis of Organoselenium Derivatives

Selenides and diselenides can be prepared by metal-catalyzed coupling reactions. Ranu et al. reported the synthesis of vinyl phenylselenides using diphenyl diselenide, copper nanoparticles, and zinc [60]. In this case the selenenylation of vinyl bromide in on-water and under ligand-free conditions produced in all cases the *E*-isomer as a major or unique product, evidencing a different mechanism if compared to that reported above for PhSeZnCl. The reaction was proposed to proceed through a copper-mediated coupling mechanism in which the sterical hindrance of the alkyl copper selenide intermediate is responsible for the observed stereoselectivity. This methodology was also applied to the phenylselenenylation of a number of aryl iodide **21** to produce the selenide **22** (Table 3). A comparison between different solvents was reported, highlighting that water is the best choice in terms of yield.

**Figure 15:**
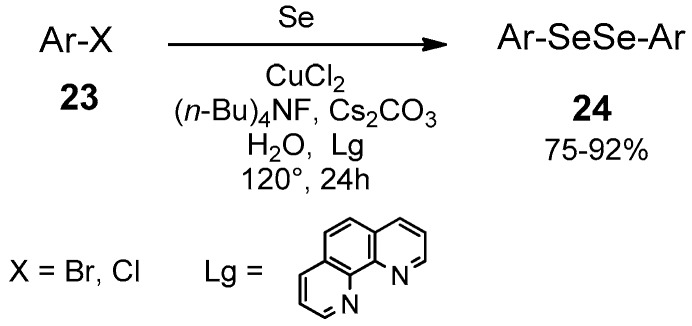
Cu-catalyzed synthesis of diselenides in phase transfer conditions.

Water can also be the medium for the synthesis of diselenides in phase transfer conditions. Using (*n*-Bu)_4_NF as phase transfer agent, Cs_2_CO_3_ as base, and CuCl_2_ as catalyst in the presence of a diamino ligand, a number of aryl bromide and iodide (**23**) was reacted with elemental selenium, producing the symmetrically disubstituted diselenide **24** (Figure 15). The reaction is particularly efficient in terms of yield, ranging from 75% to 92% [61].

The choice of solvent can also control the selective selenylation of mixed aryliodide and bromide in the presence of a diselenide and copper catalyst supported on alumina. The coupling reaction in “on-water” conditions occurred selectively in the position occupied by the iodide, whereas the same reaction performed in PEG-600 selectively involved the brominated position. This previously unobserved reactivity allowed Ranu et al. to optimize an elegant procedure for the preparation of variously substituted organo bis-selenides (e.g., **29**).

The opportune choice of a dihalo-substituted starting material (**25**), of the diselenides **26** and **28** to be used one in “on-water” conditions and the other in PEG-600 represents a good strategy for the preparation of libraries of bisselenide **29** passing through the intermediate formation of **27** [62].

**Figure 16:**
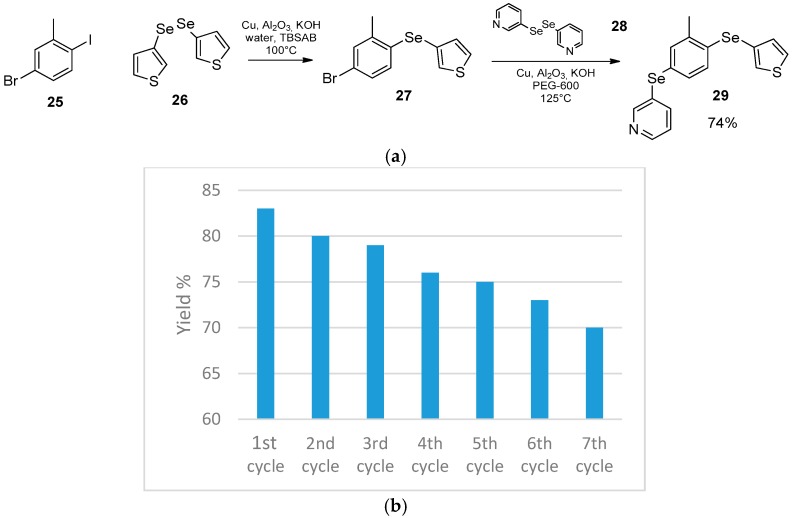
(**a**) Synthesis of the bisselenide **29**; (**b**) Reuse of the catalyst in the reaction of 4-iodoanisole and diphenyl diselenide.

Concerning the catalyst, due to its incorporation on Al_2_O_3_ it is possible to be recovered and reused, maintaining appreciable performances during the first seven cycles, as demonstrated for the reaction between 4-iodoanisole and diphenyl diselenide (Figure 16).

Very recently it was reported by Alves et al. that the solvent can also selectively direct the selenenylation of terminal alkynes toward the formation of mono-selanyl alkenes when the reaction is performed in solvent-free conditions, or bis-selanyl alkenes when the reaction is performed in water [63].

Among organocatalytic reactions, pyrrolidine has been used to catalyze a [3 + 2] cycloaddition of aziridines with isoselenocyanates using water as a reaction medium at 50 °C, producing the corresponding selenium-containing heterocycle in good to excellent yields [64].

Finally, an aqueous NaOH solution has recently been used for the synthesis of spirocyclopropyl oxindoles through a domino Michael/intramolecular nucleophilic substitution sequence using different combinations of substituted vinyl selenones and enolizable oxindoles [65], as well as for the electrochemical preparation of selenides (but also tellurides and sulfides) starting from halogenated starting materials [66].

## 5. Conclusions

As shown in this review, pure water or water/organic solvent mixtures are becoming an increasingly popular reaction medium in organoselenium chemistry. The main advantages of using this solvent as a green alternative are not simply related to the absence of toxicity and flammability, but to selectivity, efficiency and increasing reactivity, and the recyclability of the medium both in preparative stoichiometric reactions and in catalytic reactions using organoselenium catalysts.

## Figures and Tables

**Table 1:**
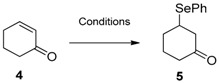
Michael-type addition to enones.

Conditions	Reaction Time	Yield %	Reference
PhSeH, water, β-CD, r.t.	45 min	82	[39]
PhSeZnCl, THF, r.t.	24 h	90	[40]
PhSeZnCl, water, r.t.	140 h	53	[40]

**Table 2:**
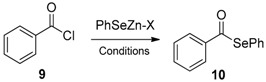
Acyl substitution for the synthesis of selenoesters.

Entry	X	Reaction Time	T °C	Solvent	Activation	Yield %	Reference
1	SePh	5‘	80	none	MW 100 watt	90	[42]
2	SePh	90‘	80	none	heating	67	[42]
3	Cl	25h	80	THF	none	25	[43]
4	Br	25h	80	THF	none	30	[43]
5	Cl	3h	rt	water	none	60	[43]
6	Br	3h	rt	water	none	70	[43]
7	Cl	5‘	rt	none	mortar	25	[44]
8	Br	5‘	rt	none	mortar	30	[44]

**Table 3:**
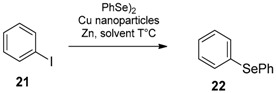
Cu nanoparticles catalyzed coupling reaction: comparison of solvents.

Solvent	Temperature	Yield %
water	100 °C	88
DMF	120 °C	78
THF	66 °C	41
EtOH	78 °C	38
MeOH	65 °C	32
